# Successful Elective Thoracoscopic Resection of Complicate Extralobar Bronchopulmonary Sequestration after Intrafoetal Vascular Laser Ablation: The Paediatric Surgeon's Point of View

**DOI:** 10.1155/2023/4959022

**Published:** 2023-08-25

**Authors:** Giulia Fusi, Agnès Sartor, Marion Groussolles, Solene Joseph, Julie Vial, Lea Roditis, Christophe Vayssière, Olivier Abbo

**Affiliations:** ^1^Department of Pediatric Surgery, Toulouse University Hospital, Toulouse, France; ^2^Department of Obstetrics and Gynecology, Toulouse University Hospital, Toulouse, France; ^3^Department of Pediatric Radiology, Toulouse University Hospital, Toulouse, France; ^4^Department of Pediatric Pulmonology and Allergology, Toulouse University Hospital, Toulouse, France

## Abstract

Few reports of laser coagulation for foetal bronchopulmonary sequestration (BPS), a rare congenital malformation characterised by the absence of tracheobronchial connection and the presence of a systemic feeding artery, have been published. Additionally, very few of them focus also on the postnatal management, with results limited and controversial. Postnatal treatment of residual malformation remains debated, hence the need to share our experience of a combined pre- and postnatal approach to complicated extra-lobar BPS. We report the case of a female foetus with the diagnosis of a pulmonary lesion. Due to mediastinal shift, unilateral compressive hydrothorax, ascites, and hydrops, a foetal treatment with ultrasound-guided laser coagulation of the anomalous vessel was performed. At birth, due to the persistence of the malformation, an elective delayed thoracoscopical surgery was performed. Prenatal laser ablation for complicated BPS is a life-saving procedure not always resulting in lesion disappearance. Thoracoscopical surgical exploration in case of persistent lesions at birth offers the possibility of a minimally invasive sequestrectomy feasible and safe.

## 1. Introduction

Bronchopulmonary sequestration (BPS) is a rare congenital malformation characterised by the absence of tracheobronchial connection and the presence of a systemic feeding artery [1].

Some BPSs, above all cases without foetal fluid effusions, spontaneously regress during pregnancy and can be managed expectantly [2], and others increase in size and may develop pleural effusions with mediastinal shifting. In these cases, mortality and morbidity increased, and prenatal treatment might be mandatory to avoid progression to hydrops, intrauterine, and neonatal death [1, 2].

To date, few studies reported in the Literature documented how complicated BPSs are successfully treated prenatally with laser ablation of the feeding artery [3–6], but very few of them focus also on the postnatal management [7, 8].

We report a case of large foetal extra-lobar BPS treated with prenatal laser ablation followed by successful thoracoscopic elective resection.

## 2. Case Report

A pregnant women was referred to our attention with a female foetus with diagnosis of the left inferior pulmonary lesion at 22 weeks of amenorrhea (WA). Ultrasonographic follow-up showed an anomalous systemic vessel of 3 mm in diameter originating from the abdominal aorta and a progressive increase in size of the lesion with mediastinal shift, compressive hydrothorax, ascites, and hydrops.

**Figure 1:**
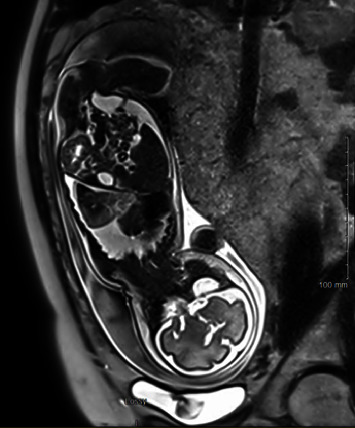
Foetal MRI at 27 weeks of gestation +3 days showing a 44 mm × 22 mm  ×  38 mm mass in the left lower lung lobe with an anomalous systemic vessel of 3 mm of diameter originating from the proximal part of the abdominal aorta, with concomitant major hydrothorax, right mediastinal shift and mild ascites.

A foetal MRI confirmed a heterogeneous mass in the left lower lung lobe and the presence of concomitant left hydrothorax and right mediastinal shift (shown in Figure 1)

A foetal treatment was performed at 26 WA.

**Figure 2:**
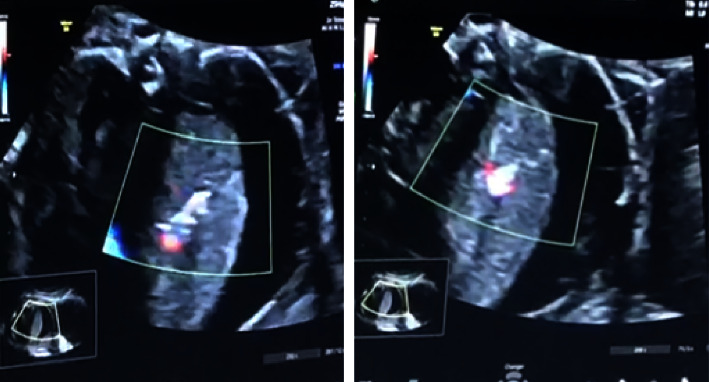
Prenatal treatment (laser ablation).

Briefly, intrauterine ultrasound-guided laser coagulation (USLC) of the feeding artery was performed under maternal spinal anaesthesia and foetal intramuscular anaesthesia. An 18 G needle was introduced percutaneously, directly into the amniotic cavity. Under ultrasound guidance, the needle was directed through the foetal thorax to the lung mass and to the main feeding artery. The 400-micron laser fibre was inserted and, at a power of 15 Watts, contacted the aberrant artery intermittently for 15 s achieving coagulation. Color Doppler demonstrated cessation of blood flow (shown in Figure 2)

The complete procedure lasted 30 minutes. No intraoperative complications were observed.

Serial ultrasound showed normal foetal growth, regression of anasarca, and stability of the size of the sequestration.

At the control MRI at 32 WA, the size of the BPS was stable with 2/3 of the mass devascularized.

The baby was delivered vaginally at 38 weeks, with a birth weight of 2550 g, poor Apgar scores, and respiratory distress that required mechanical ventilation and oxygen support.

Chest X-ray at birth showed the persistence of a solid mass in the left lower lung lobe. The patient was closely monitored, and an elective surgery was scheduled.

A preoperative chest CT scan with contrast showed stability of the extra-lobar BPS; thus, the patient underwent thoracoscopy (at 11 months).

**Figure 3:**
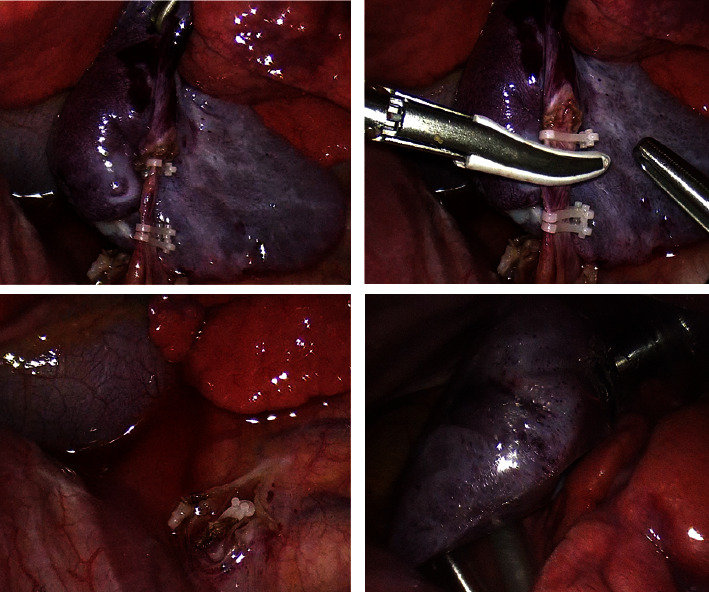
Postnatal treatment (thoracoscopical excision).

In the right lateral decubitus, a 3 mm trocar was inserted under the tip of the scapula. Two additional trocars were inserted. At the inspection, a macroscopically viable and perfused mass was rapidly identified, with the feeding vessel coming from the subdiaphragmatic aorta. The adhesions between the visceral and the parietal pleura were soft and easily removed by gentle smooth dissection. The feeding vessel was identified, closed with 3 Hem-o-Lock clip, and divided by Ligasure. The lesion was extracted intact through the posterior port with an endobag (principal steps of the procedure are shown in Figure 3)

No drain was placed. No intraoperative or postoperative complications were recorded. The patient was monitored for 24 hours, restarted alimentation immediately after surgery, and discharged at 48 hours.

Anatomopathology confirmed the extra-lobar BPS.

The patient is now 15 months old with regular growth and no respiratory symptoms.

## 3. Literature Review

A review of the English Literature was conducted on PubMed/EMBASE/Google Scholar using the following research query: “((antenatal)OR(foetal))AND((laser)OR(ablation))AND((sequestration)AND(postnatal management)AND(thoracoscopy)).” All papers were screened based on title and abstract. All papers reporting at least one case of BPS treated with prenatal laser coagulation requiring postnatal surgical treatment due to the persistence of the lesion were reviewed.

For each case, when available, the following information was collected: GA at the time of the procedure, GA at birth, postnatal imaging, type of surgery (thoracotomy or thoracoscopy), and pathologic findings (quality of adhesions). Data regarding all the cases of BPS treated with USLC with persistent lesions at birth requiring postnatal management were collected and summarised as shown in Table 1.

A review of Literature identified 6 studies reporting at least one case of BPS treated prenatally with USLC requiring postnatal management [4, 7–12] (the last two published reports were from the same institution and were summarised in the most recent published article, [7]).

Data on a total of 34 patients, including our case, were extrapolated and analysed. The mean gestational age at the time of prenatal procedure was 27 WA ± 4.24 (range 18–30). No case of antenatal death has been reported. The mean gestational age at birth was 36.83 ± 3.9 WA (range: 29–39). Regarding postnatal management, all cases included in our series required surgery after birth due to the persistence of the lesion, as indicated previously. In 16 cases, no information about postnatal radiological investigation was available, 6 received an MRI, and 11 underwent chest CT.

Postnatal sequestrectomy was performed in 34 patients (100%).

The surgical approach was open in 5 cases (15%), thoracoscopic in 4 (12%), and not specified in the others 25 (73%).

In all the thoracoscopic cases, the presence of significant, numerous, and strong adhesions was reported.

## 4. Discussion

A percentage of foetuses with extra-lobar BPS can undergo potentially fatal complications, commonly due to hydrothorax, that may cause a mediastinal shift, decreased venous return, and increased central venous pressure leading to foetal hydrops [7]. Like for the others, pulmonary congenital anomalies are important to calculate CVR (CPAM volume ratio), a useful sonographic indicator of fetuses at risk for hydrops who require close ultrasound observation and possible foetal intervention [13].

In case of complicated extra-lobar BPS, prenatal treatment might be mandatory to avoid progression to hydrops, intrauterine, and neonatal death.

Since the first case of complicated foetal BPS successfully treated with intrauterine ultrasound-guided laser coagulation of the feeding artery was presented in 2007 [13], few reports of laser coagulation for foetal BPS have been published providing preliminary evidence of potential benefit for foetal survival.

There is a general consensus about how complicated BPS can commonly prenatally be treated with satisfactory outcomes [3, 4], but a general consensus regarding the optimal postnatal management of such cases is still lacking.

In fact, essentially due to the rarity of the disease, only small series or case reports have been published in Literature to date, and very few of them focus also on the postnatal management, with results sometimes incomplete, limited, and partly controversial, including extremely good results without any need for postnatal surgery as well as cases with large residual masses after birth [3–6, 9–12].

Detailed surgical reports, with precise descriptions of the procedure, operative images, or videos describing the appearance of the thorax and the potential thoracic changes, are still lacking, hence the need to share our experience.

To the best of our knowledge, only two other authors (from the same institution) presented their experience in thoracoscopic sequestrectomy after foetal laser coagulation, focusing on operative findings, for a total of 3 case reports present to date in Literature [7, 8].

Both authors will describe the thoracoscopic appearance of the residual lesions mainly characterised by the presence of extensive severe adhesions among them, lung, and chest wall. They both detail how dissection of these adhesions was challenging with moderate bleeding [7, 8].

Discordantly, we found smooth adhesions, and their dissection was easily conducted without bleeding. In all the cases described, the feeding artery was well evidenced intraoperatively and well recognisable in its proximal part arising from the aorta. In one case, the anomalous systemic vessel was cut with Just-Right 5-mm stapler after ligature with Hem-o-Lock clip due to its huge dimension [7]. We performed the cutting using the Ligasure, with no difficulties and no bleeding, accordingly with the other cases presented [8]. We do not really explain why we found this difference but maybe it could be related to the timing of surgery (our patient was few months older than the patients operated on by the other authors).

In all cases even when the postnatal CT did not show the anomalous systemic vessel, at the moment of surgical exploration, the vascular structure was present. This was probably related to the phenomenon of revascularization of the anomalous vessel after laser coagulation and supports us in sustaining that persistent lesions at birth should undergo elective thoracoscopic resection to avoid the potential risks of leaving a residual BPS in situ. Probably, this phenomenon should let us consider the need for systematic surgical exploration of these cases.

## 5. Conclusion

BPS can occasionally present with prenatal complications correlated to foetal or neonatal death. Prenatal laser ablation for complicated BPS does not always result in lesion disappearance. Thoracoscopic surgical exploration in case of persistent lesions at birth offers the possibility of a minimally invasive sequestrectomy feasible and safe.

## Figures and Tables

**Table 1:** Summary of published cases of BPS underwent prenatal USLC with persistent lesions at birth requiring postnatal management.

Study (year)	*n*	GA at laser (WA)	GA at birth (WA)	Postnatal imaging	Postnatal sequestrectomy: *n* cases (% of total)	Age at surgery	Thoracotomy	Thoracoscopy	Type of surgery	Quality of adhesion
Cavoretto et al. [6] (2008)	8	29	38	NA	5 (63)	N/A	N/A	N/A	N/A	N/A
Rammos et al. [9] (2010)	2	30	NA	NA	2 (100)	10 days of life, first day of life	2	0	Sequestrectomy	N/A
Baud et al. [10] (2013)	1	18	29	NA	1 (100)	2 days of life	1	0	Lobectomy	N/A
Mallmann et al. [11] (2014)	5	30	39	NA	1 (20)	N/A	N/A	N/A	Sequestrectomy	N/A
Gottschalk et al. [4] (2018)	12	29	39	US, MRI, CT	3 (25)	6 months, 8 months, 6 weeks	N/A	N/A	Sequestrectomy	N/A
Zanini et al. [12] (2022)/Ichino et al. [7] (2020)	5	27	38	MRI, CT	3 of 4 (75)	5 months, 6 months,	2	3	Sequestrectomy	Strong and numerous
Our study	1	26	38	CT	1 (100)	10 months	0 (0)	1 (100)	Sequestrectomy	Soft and smooth

## Data Availability

All data generated or analysed during this study are included in this article.
